# The Proofs of the Existence of a Phthisical Contagion

**Published:** 1883-07

**Authors:** R. Shingleton Smith

**Affiliations:** Physician to the Bristol Royal Infirmary, and Lecturer on Physiology in the Bristol Medical School


THE BRISTOL

Medico-Chirurgical Journal.

JULY, 1883.

### THE PROOFS OF THE EXISTENCE OF A PHTHISICAL CONTAGION.

BY

R. SHINGLETON SMITH, M.D., B.Sc., M.R.C.P.,

*Physician to the Bristol Royal Infirmary,*
*and Lecturer on Physiology in the Bristol Medical School.*

THE contagiousness of tuberculosis is a subject of peculiar interest to the medical profession of this district, inasmuch as it was here that our late colleague, Dr. Wm. Budd, startled the profession by announcing his belief that phthisis would be found to be contagious. The grounds of his belief were as follows:—*

(*a*) Considerations based on the pathology of phthisis, as showing it to consist in the evolution and multiplication within the organism of a specific morbid matter, with a universal tendency to elimination and casting forth of the same, after the type of zymotic diseases generally.

(*b*) Actual instances in which there was evidence to show that phthisis was communicated from one person to another.

(*c*) The geographical distribution of phthisis in past

* *Lancet*, Vol. II., 1867, pp. 451—550.

and present times, and especially its great fatality now in countries which, when first discovered by Europeans, were known to be entirely free from it.

(*d*) Its much greater prevalence in low levels and amongst crowded communities, and its entire absence, unless by casual importation, at very high levels,—conditions which are well known to rule in the same directions the spread of zymotic diseases generally, and especially in that group in which, as in phthisis, the morbific matter is cast off in a liquid form.

(*e*) Its very high rate of prevalence in convents, harems, barracks, penitentiaries, &c., that is to say, under the very social conditions which are known most to favour the propagation of diseases of the zymotic group.

Among the data relating to geographical distribution the following striking facts may be here mentioned :—

1. When the South Sea Islands were first discovered phthisis did not exist there. Since the aborigines have come into intimate contact with Europeans the disease has not only made its appearance among them, but has become so wide-spread as to threaten their extermination. The contrast between the original entire immunity and the present extreme fatality is very striking, and can only be rationally explained by the importation of a new and specific morbific germ. Try every other supposition and the facts are inexplicable; make this one supposition and they are at once explained.

2. The late Dr. Rush, of Philadelphia, who made very accurate enquiries to determine the point, satisfied himself that when America was first discovered phthisis was unknown among the native American Indians. Now it is very fatal to them. The very significant contrast here exhibited between the past and present history of these two races, in respect of phthisis, is seen at once, and at the present time among the negro race in Africa in different parts of the area of that great continent.

It is well known that negroes are peculiarly liable to phthisis. Now everywhere along the African seaboard where the blacks have come into constant and intimate relations with the whites, phthisis causes a large mortality amongst them. In the interior, where intercourse with the whites has been limited to casual contact with a few great travellers or other adventurous visitors, there is reason to believe that phthisis does not exist. Dr. Livingstone and other African travellers have given us the most positive assurances on this point.

The following are the principal conclusions to which I have been led regarding phthisis or tubercle :—

1st.—That tubercle is a true zymotic disease, of specific nature, in the same sense as typhoid fever, scarlet fever, typhus, syphilis, &c.

2nd.—That, like these diseases, tubercle never originates spontaneously, but is perpetuated solely by the law of continuous succession.

3rd.—That the tuberculous matter itself is (or includes) the specific morbific matter of the disease, and constitutes the material by which phthisis is propagated from one person to another, and disseminated through society.

4th.—That the deposits of this matter are therefore of the nature of an eruption, and bear the same relation to the disease phthisis as the "yellow matter" of typhoid fever for instance bears to typhoid fever.

5th.—That by the destruction of this matter on its issue from the body, by means of proper chemicals or otherwise, seconded by good sanitary conditions, there is reason to hope that we may eventually, and possibly at no very distant date, rid ourselves entirely of this fatal scourge.

In support of Dr. Budd's statement with regard to the geographical distribution of the disease, De Quatrefages in his work on "The Human Species" says:—"In the Sandwich Islands Cook calculated the population at 300,000. In 1861 there were but 67,084. Similar facts are true of most other parts of Polynesia.

"Two naval surgeons have been able to throw light on this melancholy problem. They found that tubercles were invariably found in the bodies submitted to examination. Almost all the Polynesians suffer from an obstinate cough, and in eight cases out of ten tuberculosis follows these bronchial catarrhs.

"Now phthisis does not appear in the list of diseases drawn up by the old voyagers. Have we then imported it into these islands? Developing in a new region, in a race to whom it was formerly unkown, this disease assumed a more terrible form."

The researches of later times have given much support to a view which has long existed in a smouldering unstable condition. The most recent discovery, that of Professor Koch, of a definite distinguishing bacillus of characteristic form and size, found in tubercular matter in the body and in tuberculous excreta, capable of being inoculated, capable also of purification by successive growths by a process of artificial cultivation, these facts have given new life to an idea which appeared to be almost dead, have given an impetus to the doctrine of contagium vivum which is not likely to be short lived.

Even as long ago as the times of Galen consumption was supposed to be contagious, and antiseptic treatment of a primitive kind was first attempted when Galen ordered his patients to inhale the sulphurous and other exhalations from the crater of Etna.

It is only about twenty years ago that Buhl began to teach, apparently as a new idea, that acute tuberculosis is of an infective nature, the disseminated grey granulations being supposed to be the result of absorption of some poisonous material from a caseous focus originating in some inflammatory or scrofulous action.

*Experimental Evidence.*—Villemin, in 1865, demonstrated the possibility of inoculating animals with tuberculosis. He found the disease could be readily induced in dogs and rabbits by the inoculation of little bits of tubercle inserted under the skin. Hypodermic injection of small quantities of the sputa of consumptive patients gave the same result.

Villemin's* experiments showed conclusively the possibility of inoculating tubercle from men to the lower animals, and from one animal to another. These results were fully corroborated by Simon,† who found that both the yellow and the grey tubercle are inoculable from man to the rabbit, and from rabbit to rabbit. He concluded that whether it be called tubercle or not the action must be allowed to be specific. A commission of inquiry appointed by the Pathological Society of London to report on Simon's specimens "thought the tuberculous nature of the specimens was beyond reasonable doubt." So also a French commission gave Villemin credit for revealing a fact of the highest interest, the transmission of phthisis by the inoculation of tuberculous matter. Bizzozero, Colin, Biffi, and Mantegazza have confirmed these results, and satisfied themselves that the growths in the viscera

* Villemin—*Lancet*, I., 1867, p. 582, 641; II., 1867, p. 774.
† Simon—*Lancet*, I., 1867, p. 367.

of rabbits inoculated with human tubercle are really tubercles.

The researches of Villemin and Cohnheim established the fact that true tubercle may always be inoculated, and this became established as the test by which true and false tubercle might be differentiated. The capacity of a morbid product, when introduced into the body of a rabbit or a guinea pig, to produce tuberculosis in the animal demonstrates the nature of that morbid product: what produces tuberculosis is tubercle, what fails to produce tuberculosis is no tubercle. The following paragraph, written in May, 1880,* is quite prophetic of recent discoveries:—"Those who are convinced that all contagious virus is due to a parasite will, of course, not hesitate to believe that the tubercular poison is corpuscular, and that, in a not very distant future, this corpuscle will be demonstrated in the tuberclenodule and in scrofulous products. Until, however, this goal is reached there remains no other test for tubercle than that of infection." The writer of this paragraph scarcely suspected that in two years afterwards the examination of sputum in order to establish the diagnosis of phthisis by ocular inspection of the Bacillus tuberculosus would be an every-day occurrence in the wards.

Then came the experiments of Lebert, Burdon-Sanderson, Andrew Clark, Wilson Fox and others, who at first appeared to establish the fact that what was called tuberculosis in animals might be produced by the inoculation, not of true tubercle only, but of any of the products of inflammatory action, or even of putrid substances or by foreign bodies such as a seton. Doubts were thus thrown on the nature of tubercle itself, and it appeared to be

* *Brit. Med. Jour.*, p. 704.

difficult to say what might be true and what pseudo tubercle—the barrier between inflammatory action and a specific tuberculosis appeared to be giving way, and so it got to be assumed that most of the changes going on in the lungs in phthisis were of an inflammatory nature, and that the rôle of the specific substance, the grey granulation, was far more limited than had been supposed. The experiments of Dr. Hippolyte Martin, of Paris, caused a swing of the pendulum in the opposite direction, and appeared to re-establish the existence of a specific virus without which tuberculosis could not be produced.*

Dr. Martin conducted two sets of experiments to prove the infectiousness of tubercle. With the greatest antiseptic precautions he injected into the peritoneal cavity of guinea pigs irritating substances of vegetable and animal nature, such as pepper, cantharides, fragments of sarcoma, carcinomatous mamma, particles from the caseation of a sarcomatous tumour, and other non-tubercular matter, with the result that in most instances the animals remained quite healthy, and when killed had no disease of the viscera. In other cases he showed that foreign bodies could set up ordinary inflammatory action, the products of which could not be anatomically distinguished from true tubercle, which always followed inoculation with true tuberculous matter. These secondary tubercles, after inoculation with tuberculous matter, had the distinguishing characteristic of increasing infectiousness from successive inoculations. Hence he concludes tubercle is an infective malady, owing to the presence of some morbific agent, peculiar to it, but undetermined.

The most remarkable inoculation experiment on record, and one which has not attracted so much attention

* *Lancet*, Aug. 5, 1882, p. 172.

as it appears to deserve, is the only recorded case of experimental inoculation in the human subject:—

"Demet, Paraskeve, and Zallonis, in Syra, Greece, were not only successful in transmitting the disease to rabbits by inoculation with the sputa and blood from a man affected with phthisis, but they ventured on the experiment of inoculating a human patient whose history gave no indications of tuberculous taint, and whose lungs were found to be healthy, but who was suffering from gangrene of the big toe, due to obliteration of the femoral artery. A fatal termination was inevitable, and sputum from a man who had abscesses in his lungs was inoculated in the upper part of thigh. Three weeks afterwards there were evidences of disease at the summit of the right lung, and at the time of death, thirty-eight days from date of inoculation, it was found that the upper lobe of right lung had seventeen tubercles in first stage, two of the size of a lentil, the others as large as a grain of mustard. Two similar tubercles existed at the apex of the left lung. Two tubercular masses were found on the convex surface of the liver."*

Numerous other observations show that injection of tuberculous matter from man, from the ox, or from the milk of tuberculous cows will give rise to tuberculosis in pigs, sheep and goats, rarely a positive result in rabbits, a negative result with dogs and cats. Later Viseur, at Arras, showed that cats were not exempt from tubercle induced by the eating of tuberculous matter.

"Klebs found that rabbits and guinea pigs were very susceptible to the influence of milk from tuberculous cows, and he accidentally induced the disease in a dog by feeding it with the milk of a phthisical cow. The vital tenacity

* *Med. Chi. Review*, Oct., 1874.

of tubercle virus appears to be remarkable, as shown by these inoculation experiments: it preserves its potency through three or four generations. Inoculations will succeed with matter from a subject which has been dead 36 hours, and with sputa which has been dried for 20 days. If thoroughly boiled its vitality is destroyed, and Klebs found that the action of alcohol deprived it of its potency."*

"Chauveau, of Lyons, in 1868, administered to three healthy calves about thirty grammes of tuberculous matter from an old phthisical cow: the caseous, puriform, and cretaceous mass was pounded in a mortar and dissolved in water, and then administered from a bottle.

"Fifty-two days afterwards the miserable aspect of the three infected creatures contrasted in the most extraordinary manner with the thriving condition of the non-infected one which had been kept under similar conditions for comparison. The autopsy revealed the most perfect lesions of general tuberculosis, the mesentery and intestines being extremely involved. He concludes:—

"1. The experiments prove that bovine animals contract tuberculosis by digestive injection.

"2. They place beyond a doubt the fact as to the virulence and contagious property of tuberculosis.

"3. That the digestive canal constitutes in the bovine species, as in man, a channel of contagion readily disposed for the propagation of tuberculosis, and which may be more frequently the mode of access than through the pulmonary organs."†

Other observers established the existence of a definite virus in other ways: dogs and various animals were made to breathe an atmosphere in which the sputa of consump-

* *Med. Chi. Review*, Oct., 1874. † *Ibid.*

tive patients were diffused in the form of spray, whilst they made others respire air through which they diffused atomised liquids impregnated with non-tuberculous substances. It was found by Tappeiner,* and others, that in all cases where real tuberculous matter was used tubercles were found in the lungs, kidneys and other organs, the abundance of the tubercles being generally proportionate to the length of the experiments and the frequency of the inhalations. In those cases where non-tuberculous matter had been sprayed into the air no tubercles were found. Carrying the idea a stage further, M. Giboux caused rabbits to inhale the air expired by consumptive patients, and he found that at the end of about three months the rabbits emaciated and at last died from general tuberculosis, whereas other rabbits which were made to inhale similar air filtered through tow charged with carbolic acid showed no impairment of health, and when killed were found to be free from tubercle.

All these experiments point to the conclusion that there must be some special infecting virus in tubercle, and it is now five years ago that Professor Klebs believed that he had demonstrated a necessity for the existence of a microphyte, monas tuberculosus, to which he presumed the contagiousness of tubercle was due; and thereupon Dr. Schüller, of Greifswald, and others advised the more active antiseptic treatment of the disease.

In spite, however, of the suspicions and speculations of various thinkers, and in spite of the abundant evidence from inoculation, evidence amounting almost to mathematical demonstration, no one had as yet succeeded in detecting by ocular evidence any microphyte which might be looked upon as the specific germ by which the disease

* Virchow's *Archives*, vol. lxxiv., 1878.

might be inoculated and by the dissemination and growth of which it might be supposed to spread. *Professor Koch, of Berlin, has recently given to the world the most striking corroboration of the truth of the theory which hitherto had not received general acceptance. Koch, having previously demonstrated the existence of the bacillus anthracis as the cause of splenic fever, and of various other bacilli in cases of septicæmia, set himself the task of discovering the specific germ which so many authors believed must exist, although its presence could only be proved by indirect evidence. By special methods of aniline staining Koch was at last able to show that in all true tubercles found in cases of general tuberculosis a peculiar micro-organism could be seen. The bacilli of tubercle were found to be delicate rod-shaped bodies, varying in length from quarter to one half the diameter of a blood corpuscle. They are found in all cases within the giant cells of the grey granulation, and are most numerous in recent and advancing tubercle; they become fewer in proportion as the tubercle gets older, and finally disappear on the healing of the tubercular ulcer. The expectoration also was found to contain multitudes of these bacilli, whilst none could be discovered in the sputa of non-tubercular patients. He also found that the bacillus of tubercle required, in inhabitants of the temperate zone, about the warmth of the animal body for its growth and increase, that in fact the bacilli did not increase in numbers in the sputum whereas the putrefactive bacteria would rapidly increase.

A special dye was discovered by which the bacilli could be isolated from all other substances, and thus readily demonstrated by its colour. The process was afterwards

* Berliner Klinische Wochenschrift, No. 15, 1882.

improved by Ehrlich; and various other methods, more particularly that of Heneage Gibbes, have come into operation. The principle of all these methods is the same, viz., to stain the bacilli of one colour and the ground substance of another, by which contrast the minute germs can be readily seen even without the highest powers of the microscope. Professor Koch did not stop here; having shown the existence of a definite germ, having described its characteristic appearance and its difference from other bacilli which accompany it; having shown that by staining agents it may be differentiated from other similar bodies, and that it requires a special temperature for its growth and multiplication, he then endeavoured to prove that this visible germ is the germ by which the infection of tubercle is conveyed. His attempts were crowned with complete success; he was able to cultivate the bacilli artificially through many successive generations; after transference from one soil to another a great number of times the original tubercle matter was at length got rid of, and nothing but the bacilli left behind. He then ascertained that inoculation with these bacilli, isolated from all the original matter with which they were associated, was followed by reproduction of the parasite, and the induction of tuberculosis, as certainly as when the tuberculous matter itself was inoculated. Further he found that filtration of the fluid containing the bacilli through porous clay destroyed its virulence, the fluid which passed through became innocuous, whilst the substance which refused to pass the filter would readily give rise to tuberculosis.

The method of cultivation of the bacilli adopted by Koch is not an unimportant part of his discovery, inasmuch as it enables him to isolate any particular form of growth from others which may be accidentally associated with it. The pure cultivation of any particular pathogenic bacterium is effected by adding three to five per cent. of gelatine to the infusion or other substance with which he is experimenting; in this way growth takes place on a *solid* soil, which can be clarified, and will so allow of constant observation as to what is going on upon or within it.

If any accidental growth occur it only spreads at one point, and fresh material can be inoculated from a pure part without risk of contamination.

The bacilli have been found by Dr. Koch in the following cases:—Eleven cases of miliary tuberculosis, twelve cases of cheesy broncho-pneumonia, one case of tubercle in brain as large as a hazel nut, two cases of intestinal tuberculosis. Three cases of freshly extirpated scrofulous glands were examined, in two of which bacilli were found enclosed in giant cells; and in two out of four cases of synovial degeneration of joints they were present in small numbers in giant cells.

Among the lower animals ten cases of perlsucht, three cases of bronchiectasis in cattle, a cheesy gland in a pig, a tubercular hen, three monkeys which died of spontaneous tuberculosis, nine guinea pigs and seven rabbits which had spontaneous tuberculosis, and 172 guinea pigs, 32 rabbits, and five cats which had been infected with the disease; in all of these the tubercle-bacilli were found.

In this way the constant presence of a distinct organism in association with a distinct pathological process was clearly demonstrated.

On the other hand it was found that inoculation with material which did not contain the living bacilli was not followed by tuberculosis. In all cases when the material inoculated contained the living bacilli the result was positive, but when the material inoculated did not contain bacilli a negative result was obtained, the animal remaining healthy in spite of inoculation. Experiments were performed with the contents of a scrofulous gland, and with degenerated synovial membrane, neither material containing bacilli; and also with the tubercular lung of a monkey, some parts of which had been dried for two months, and some kept one month in alcohol, and in no instance did tuberculosis follow.

That the disease following inoculation was not due to accidental causes independent of the bacilli was shown by the fact that the artificial disease differed from the spontaneous one: such a plentiful crop as occurs after inoculation never develops spontaneously in so short a time; and again in the natural disease the bronchial glands become enlarged and cheesy and then the lung is affected, whereas in the artificial disease the glands nearest to the seat of inoculation (generally in the abdomen) were first enlarged, and the spleen and liver were specially affected.

In order to prove that the bacilli were the real and only cause of the infection it was necessary to proceed a step farther, and to isolate them from all their surroundings by the process of pure cultivation on a solid substratum of gelatine. The ordinary gelatine would not do for the purpose, inasmuch as it was found that these bacilli require a temperature approaching that of the body for their growth, and at this temperature the gelatine cultivating preparations become fluid. The material which was found to answer the purpose the best is blood serum which has been rendered solid by heat, but which has not been exposed to so high a temperature as to become opaque. It was found that about the tenth day after inoculation of this solid blood serum with any tuberculous material small points and scales became evident, which slowly spread, and were seen to consist of tubercle-bacilli. After about fourteen days some of these bacilli were transferred to fresh blood serum, and this process was repeated several times. Thus the original material was entirely got rid of, and only the bacilli developed from it remained; and now it was found that inoculation with the pure bacilli produced tuberculosis with the same certainty as did the inoculation of tuberculous material containing living bacilli.

Mr. Watson Cheyne, in his report to the Association for the Advancement of Medicine by Research, confirms in every detail the conclusions given by Koch. He says:—

"In man we have the disease termed acute miliary tuberculosis, which resembles in every respect—histological structure, tendencies and presence of bacilli—the acute tuberculosis produced in animals by the inoculation of tuberculous material. It will be admitted that the two diseases are identical, and as they are identical their cause must be the same, viz., the tubercle bacillus. We may therefore say definitely that the tubercle bacillus is the cause of acute tuberculosis, and that scrofulous glands, degenerated (strumous) synovial membranes of joints, phthisical lungs (in short, all those materials obtained from man which, inoculated into animals, produce acute tuberculosis), contain in them bodies (bacilli) which, if they entered the circulation in sufficient numbers, would give rise to tuberculosis. It has been demonstrated by several observers that probably in all cases of acute tuberculosis a place can be found where these bacilli get into the circulation."*

* *Practitioner*, April, 1883, p. 307.

Experiments on the lower animals appear to establish the fact that phthisis, being the result of a specific poison, the tubercle bacillus, must be classed amongst the contagious diseases. What evidence can be adduced from the clinical side in support of this view?

*Clinical Evidence (Animals).*—As regards the lower animals it appears to be generally admitted that tuberculosis is contagious. Fleming* states that the disease tuberculosis is common in animals, especially in confinement, and that the bovine species are most affected. Cruzel says that in one hundred old oxen fattened and slaughtered for food at least one half will have the lungs more or less tuberculous, and further that the disease is communicated by the expired air in low badly ventilated buildings, and that it is rare that the breath of bovine animals affected with phthisis has not a remarkable odour from which the disease may be diagnosed. Viseur, of Arras, in 1868, pointed out the persistence of the malady in certain establishments. Zundel also found this in Alsace; he observed that healthy animals soon became affected in particular stables. A remarkable illustration of this is that given by—

"M. Grad, Alsace. Every year for five years one of his cattle died of phthisis, and what is very curious, said the farmer, this always happens in the same stall. The sixth died, a healthy cow placed there by M. Grad. The stall was then rebuilt, after thorough disinfection, and no more tuberculosis appeared."

Numerous examples of phthisis from inhabiting a contaminated stall are recorded by various writers.

Further instances are recorded of contagion from the bovine and feline to the human species, or *vice versa*:—

* *Med. Chi. Review*, Oct., 1874.

"Cullimore reports that a strong healthy dog lapped up the sputum of a tuberculous man and died shortly of phthisis."*

It seems highly probable that contagion may spread by the milk of high class cows which are very subject to tubercular disease.

Mr. Law, of Cornell University, writes:—

"In a case that recently came under my notice in Brooklyn, New York, a family cow was found in an advanced state of tuberculosis, and the owner and his wife were evidently rapidly sinking under the same malady. In another case reported to me, a family cow, supposed to be suffering from the lung plague, was found to be afflicted with tuberculosis instead, and the owner's wife (a consumptive), who had been making free use of the milk warm from the cow, was persuaded to give it up, and underwent an immediate and decided improvement. It is for infants and adults who are somewhat infirm or out of health, or whose surroundings are not of the most salubrious kind, that the danger is greatest; but this embraces such an extended class that the moral interests involved are almost illimitable. The destruction of infancy and wasting of manhood from this cause is unquestionably far greater than has been hitherto realised."†

*Clinical Evidence (Man).*—The facts with regard to contagiousness from man to man are thus given by Petersen, in 1870, who sums up the results of his inquiries and observations in the following propositions:—

"1. That a contagious origin of some cases of phthisis cannot on sufficient grounds be denied.

"2. That phthisis caused by contagion is in general of a very dangerous and inflammatory character.

* *Brit. Med. J.*, May, 1880. † *Ibid*, Sept., 18, 1880.

"3. That it must justly be considered hazardous to lie in the non-disinfected bed of a phthisical patient, and to be habitually in too close contact with such a person.

"4. That this danger in Denmark seems to be greatest in the warm period of the year."*

My own experience on this point is limited to an occasional case in which the husband appears to have taken the disease from the wife, or the wife from the husband.

About two years ago I was consulted by a young married man whose wife, much older than himself, had recently died of tubercular phthisis after an illness of about twelve months. His family history was good, father and mother both living, over 60 years of age, and no history of phthisis in his family. His own health had been good till six months after the death of the wife, when he noticed a little cough and expectoration in the mornings with occasional streaks of blood. Hæmoptysis in small quantity occurred in October, November, December, 1880, twice in February, 1881, and in March there was a little crepitation at the left supra scapular region. In March, 1882, he had quite recovered: there were no physical signs of disease, but patient was seldom free from cold, and had lost more than a stone in weight. He is now (May, 1883) quite well.

Another case which has come to my notice is that of a husband and wife in a country village reputed to be healthy, who both died within a day of each other of tubercular phthisis, six years after their marriage. The wife, aged 30 at time of death (in August, 1882), was one of five sisters, whose father died of consumption in 1871. She had never been very strong, and at the age of 18 was supposed to be consumptive, at the time when her

* *Med. Chi. Review*, July, 1870.

father was laid up with lung disease. After her marriage she seems to have been quite well, and stronger than before, with no suspicion of any disease of the lungs. Three years after their marriage the husband, then aged 26, got a succession of colds, and was never well afterwards. Two years after the commencement of the husband's illness the wife caught cold, spat blood, and then rapidly failed in every respect. She was in constant attendance on, and cohabited with, her husband during the whole period of his illness.

"A remarkable series of cases occurred at the Bristol Royal Infirmary in 1878, which have been quoted by Creighton as cases of infective tuberculosis occurring in an epidemic form in the human subject similar to the well known bovine tuberculosis. Three cases occurred in a reformatory school, two of them were patients of my colleague, Dr. Spencer, and one was under my care. They were all presumed to be cases of enteric fever, but they had no very decided typhoid symptoms. All died, and after death were found to have general tuberculous meningitis and intestinal lesions, but not quite of the enteric character. A few small ulcers existed in the ileum, but there were none in the large intestines. In one a few miliary tubercles surrounded a caseous nodule in the lung, which was presumed to be the focus from which tubercle infection had emanated under the influence of a typhoid pyrexia."*

Mr. G. A. Brown, of Tredegar, gives me the following history:—"A family named G. Husband a delicate man, with a doubtful history of phthisis in his family. Wife a strong, hearty woman, with no history of phthisis in any member of her family. Their family consisted of two

* *Bristol Royal Infirmary Reports*, Vol. I., p. 167.

daughters and one son. The son died, aged 18, from an accident. The younger daughter died in October, 1879, from phthisis, aged 18. The father shortly afterwards had a bad attack of bronchitis. He subsequently became phthisical, and died September, 1880, aged 50, of phthisis. The mother, during her husband's last illness, complained of cough, and spat blood. She rapidly lost flesh, and died of phthisis in April, 1881. The elder daughter is still a strong, hearty woman."

The facts suggest two questions:—1. Did the younger daughter inherit from the father's side a phthisical taint, which developed subsequently in the father himself? 2. Was the mother's a case of phthisis communicated from husband or daughter?

Mr. Bernard Kendall, of Clifton, gives me a history of two cases of apparent contagion:—" C. K., fifth son in a family of thirteen children (of whom eight are now living, the eldest being 60 years of age) married a lady in delicate health, and who was constantly obliged to winter by medical advice at Torquay, or other places in the South of England. They lived together for about six years, when the wife died of phthisis, and the husband, who was supposed to have contracted his disease from her, died about two years afterwards from the same disease. One other member of the family died of phthisis, a younger brother, who was in the mercantile service, and who contracted the disease in the China seas."

" M. W. died of phthisis, and was closely attended by his wife, who had previously enjoyed good health. She is supposed to have contracted the disease from her husband, and she died last week of phthisis."

Numerous isolated cases of apparent contagion are recorded by various observers, and the enquiries being made by the Collective Investigation Committee of the British Medical Association are likely to bring out some interesting results.

Macaldowie, from a total of four hundred patients in the North Staffordshire Infirmary, found four cases in which the wife became infected from a tuberculous husband.*

Dr. Murrell reported a case whose wife was infected by her husband.†

Dr. H. Weber has adduced evidence in a series of cases where the several wives of consumptive husbands fell successively victims to tuberculosis.

Leudet ‡ gives an account of fifty-six families, of which in fifteen the husband was tubercular at marriage and the wife then healthy, and in forty-one the wife was alone affected. Of the first fifteen, in five the wives became affected, two having had relatives dying of consumption, and a third not becoming affected till ten years after her husband's death. Of the other series, only three husbands became affected, and of these one had lost a sister from tuberculosis. He concludes as follows:—

"1. Wives contract tuberculosis more readily from their husbands than husbands from their wives.

"2. Wives who are not themselves affected may give birth to children who die of phthisis.

"3. Marriage hastens the fatal termination.

"4. Tuberculosis may often develop among different members of the same family at short intervals without hereditary predisposition."

In warm climates opinions are more decidedly in favour of contagion. In Italy quarantine regulations used to be

* *Lancet*, II., 81, p. 825. † *Ibid*, I., 80, p. 802.
‡ *Lond. Med. Record*, Jan. 15, 1883.

enforced regarding deaths from phthisis; innkeepers and others have levied a heavy tax for disinfection of rooms where consumptives have been. In India such patients are said to be dangerous as lepers.

Dr. Reginald Thompson adduces evidence that a special form of phthisis exists which he calls "Infective," a disease with peculiar symptoms and signs of its own, approximating it closely to those of pyogenic or infective pneumonia. The observations embrace a period of 10 years, and include 25,000 patients; in a series of 15,000 cases only fifteen of them were of this infective kind—one per thousand. The characteristic features are:—A slight hacking cough the earliest symptom of infection, symptoms of constitutional poisoning, rapid emaciation, rigors, occasional hæmoptysis, physical signs bilateral, local disease disproportionate to the amount of constitutional symptoms, physiognomy not that of phthisis, skin sallow not pale, with history of contagion, and absence of family history.*

The weakest chain in the argument appears to be the evidence, or rather the want of sufficient evidence, derived from clinical experience. How comes it that with the special infective germ so widely distributed as it must be that we do not all die of tuberculosis? The marvel is not that so many are affected, but that so many escape. Clearly there must be a special nidus in which alone the tubercle bacterium will prosper: like other bacteria a special soil is necessary for this particular form. The researches of Tyndall and others have shown that bacteria of putrefaction grow very unequally in different fluids: one important element in their growth is fitness of soil. The same condition of things exists with regard to all the zymotic diseases: all persons are not equally susceptible

* *Lancet*, Nov. 6, 1880, 726.

to the action of the poison of variola, scarlet fever, typhus, &c. We can only account for the escape of the many, by supposing that there are certain conditions of health in the few affected ones, which so degrade the vitality of the tissues as to render them a fit breeding ground for this particular form of bacterium, and that only under these conditions will the parasite germinate and prosper. On the other hand there are certain mechanical difficulties in the way which may also account for the only occasional growth of these organisms in those who appear to be fit subjects. It is probable that the lungs are the more usual mode by which the germs gain access to the body. Now, it has been assumed by Lister, and proved by Prof. Tyndall,* that the air which passes along the respiratory passages becomes purified in its transit, and is absolutely aseptic in the air cells and finer air passages: hence it follows that any particles suspended in it will be arrested by the mucous membrane and the larger tubes, and the bacilli will therefore be unable to reach the deep recesses of the lungs: when so arrested ciliary action will gradually cause them to travel back towards the trachea, and they will ultimately be ejected in the bronchial mucus discharged by coughing. Koch found in his cultivating experiments that, under ordinary conditions simulating those within the body, the bacilli undergo little or no change for about ten days; meanwhile long before germination has taken place they will be too far away from the lungs to effect any mischief. It is true that particles of carbon and other foreign bodies, such as cinnabar, do and may be made by experiment to reach the ultimate recesses of the lungs, but it is probable that this mechanical difficulty in transit may be one of the causes

* Tyndall, *Floating matter of the air*, pp. 36, 37.

why so many persons escape the action of these widely distributed germs.

If mere debility, a mere want of resistance to outside influences, a mere degradation of vitality of the tissues constituted the reason why one begets the disease and another does not, it would be difficult to account for the only occasional cropping up of phthisis as a direct sequel to various acute diseases more particularly of the lungs. It is a familiar fact that cases of chronic pleurisy of simple inflammatory origin, although in general aspect simulating phthisis, are yet essentially different in their nature and results; the specific virus is wanting, and the patient slowly gets well after a very protracted illness, which can scarcely be distinguished clinically from some forms of tubercular phthisis. Again it has been a matter of constant remark that "cold" is powerless to excite tubercle. The soldier, exposed to all the inclemencies of a campaign, sees phthisis disappear from the ranks, while in the comfort and warmth of garrison life he finds his comrades decimated by its ravages. The sailor, again, threatened with tubercular lung disease, finds that the open air life at sea, with exposure to all weathers and in all latitudes, soon restores the failing health and removes the disease which may have already commenced. The germ theory of tuberculosis also accounts for the fact that tubercle loves low-lying places, and is not met with at certain altitudes: just as Professor Tyndall could safely open some of his hermetically sealed tubes with organic infusions at the top of some of the alpine peaks without risk of introducing germs from the pure air around, so the pure air of these altitudes is little likely to give rise to a purely zymotic disease. Again, the warmth of the tropics is likely to be favourable to the growth of the bacilli, which accounts for the rarity of the disease at the poles, the increase of intensity towards the tropics, and its exceeding malignancy there. Tubercle, again, finds its habitat in crowded places, and is intense in direct proportion to the concentration of the population, as in prisons, barracks, factories, &c. In illustration of this point may be mentioned the fact that phthisis was found to be three times more prevalent in Millbank than in London generally, and the prisons of France and Algeria were found to show similar results. Mere crowding in all parts of the world on the other hand, and re-breathing the same air as in the snow huts of the Greenlander, has not been shown to give rise to phthisis.

The absence of a frequent occurrence of direct contagion will scarcely be considered a powerful argument against the formidable array of facts in favour of the contagiousness of phthisis. It is highly probable that a more careful observation of the conditions surrounding the onset of the disease will show that a possible direct contagion is more frequent than is generally supposed, or has yet been shown to be. Every one will remember the long debated question as to the contagiousness of typhoid: no one now doubts the existence of a definite typhoid poison to which alone when introduced to the body under certain favourable conditions the disease is due: we cannot always trace the mode of entrance, but we admit that the poison must have been introduced. Just as the typhoid poison is discharged by one particular excretion, so in phthisis the poison may be given off by one excretion only, and this not in a form which is likely to gain access to the bodies of others whilst the germs are still active. There is however one way in which self-infection from one part of the body to another is very easy, and it having been shown that tuberculosis in animals is easily induced by artificial feeding with tuberculous matter, is it not probable that the intestinal lesions of chronic phthisis are the result of self-infection by the swallowing of the secretions from the lungs?

Mr. T. F. Edgeworth reports to me a case of chronic diarrhœa, with disease in the lungs, which is clearly of a tuberculous nature; the patient never expectorates anything, but on extracting the mucus from the throat after a cough the product is found to contain numerous bacilli, and the fæcal evacuation also contains bacilli, with the characters of those found in tuberculous sputum.

Dr. Davey, of Clifton, informs me that insane patients rarely expectorate, but he was not able to form an opinion as to whether intestinal ulceration is more frequent in cases of phthisis found in the asylums for the insane than in those found elsewhere. On my mentioning this topic to Dr. A. Law Wade, of the Wells Asylum, he examined the recent records, and found that since July, 1882, there had been 14 deaths from phthisis, and in six of these there were tubercular ulcers in the intestines; in two the lining membrane of colon was thickened and congested throughout; in two there were patches of deep congestion, which did not appear to have proceeded to actual ulceration of surface, both of these were of short duration, one was a case of acute tuberculosis, and the other of phthisis with acute pneumonia.

Some confirmatory evidence as to the contagiousness of phthisis has been adduced by Mr. Thomson in his work on the Germ Theory of Phthisis, verified and illustrated by the increase of phthisis in Victoria. His statistical labours have shown that the mortality from phthisis in Victoria is greatly on the increase, viz., from 7.9 per

10,000 living between the ages of 15 and 25 in 1871, to 9.9 per 10,000 living between the same ages in 1881, or taking all ages from 11.49 per 10,000 in 1871 to 13.90 in 1881. Mr. Thomson regards "all idea about climate conferring an immunity from the disease as the most pernicious doctrine that could be inculcated." He maintains that the disease is never of climatic origin, nor is it due to dampness of soil or depressing circumstances of life: thus he regards the prevalence of phthisis amongst servant girls as due solely to the inhalation of the germ laden dust of the bedrooms they have to sweep out.*

Dr. W. T. Belfield † alludes to the argument against the infectiousness of tuberculosis founded on the fact that we do not all die of this disease. He says, "The general principle—the survival of the fittest—seems to have been for generations at work in eradicating the disease from the human family by removing those members of it susceptible to tuberculosis; the great majority of us now living are as safe from tuberculosis as most dogs are from anthrax." He also states that "one after another, the German and French pathologists (who are not infrequently clinical teachers as well), honest in their previous conviction that the communicability of tuberculosis was not proven, honestly recorded their convictions as succeeding proofs were furnished . . . . so that three years ago Cohnheim said, 'To-day there scarcely exists a pathologist who would deny that tuberculosis is a communicable disease.'"

The supreme question now before the medical world is, not whether tuberculosis is infectious, but whether the bacillus of Koch is the infective agent. The two questions are quite independent, the former established, the latter

* *Med. Record*, Feb. 15th, 1883.

† *New York Med. Record*, March 10th, 1883.

awaiting confirmation. That confirmation has now been given by Cheyne.*

I cannot refrain from quoting the following suggestive remarks of Dr. Solomon Smith.† He says: "Here we have an instance in which the contagion habitually passes over the strong to attack those who are fitted for it either by constitution or by preceding disease, or by reduced surroundings, and even when it has picked out a feeble member of the flock and attacked such an organ as the lung, is usually impotent against those portions whose functional activity is greatest, and is prone to affect those in which renewal of air and removal of exfoliated epithelium are least perfectly performed, such as parts which have been inflamed or tied down by previous thickening of the pleura, and especially the comparatively unused apices of the lungs. When we see the chest of a phthisical patient covered with patches of cloasma, the thickened and unremoved epithelium infiltrated by the spores and mycelium of the microsporon furfur, we may carry the mind's eye inwards and think of the apices of the lungs and the similar processes going on within them, their air cells, little moved by the feeble respiratory efforts of the patient, gradually plugging up with exfoliated epithelium, matted together by the growth of tubercular bacilli, which, although carried in far greater number into those portions of the lung whose functional activity is greater, have only been able to take root in the half-dead epithelium of the stagnant apicial air-cells."

Additional evidence in favour of, and in demonstration of the existence of a contagion in phthisis, has been adduced in the investigation of the question as to the diagnostic and prognostic value of the bacillus tuberculosis

* *Practitioner*, April, 1883. † *Lancet*, August 26th, 1882.

in the sputum and elsewhere. Various observers have worked energetically in this direction, and the most important results are the following:—

Balmer and Fræntzel* have given an account of 120 cases of phthisis in which the sputa were examined. The bacilli were found in all. They believe that the number and degree of development of these are of considerable prognostic value, that the more rapid the process of lung destruction the larger is their number, it being greatest towards the end of the disease. They found that in the rapid cases the organisms were not only numerous but large, well developed, and spore-bearing, but on the other hand, when the disease was arrested or making very slow progress, they were small, presented no trace of spores, and were always few in number.

They sum up their conclusions thus:—

"1. A definite prognosis may be made from the number and stage of development of the bacilli in sputa.

"2. The number of bacilli in sputum increases proportionately with the advance of the destructive process in the lung, and attains its maximum as the end approaches.

"3. Distribution of bacilli is not in all cases the same. Sometimes they are evenly scattered and sometimes are in groups.

"4. Their appearance is not constant. They are often small, ill developed, and without spores, and small in number.

"5. Such bacilli are found when the disease is progressing but slowly or has been checked.

"6. Bacilli are large and rich in spores in all rapidly advancing cases with high temperature, night sweats, &c.

* *Berl. Klin. Woch.*, 1882, No. 45.

"7. In every case in which the bacilli were plentiful there was high fever, and the converse was also true.

"8. The difference in quantity of bacilli in the fluid of a cavity and that in the lung tissue surrounding the excavation was very marked, numerous in the former, scanty in the latter.

"9. From this it appears that the sputum affords a more favourable place of growth than does the still living lung tissue.

"10. We may not ascribe the rich development of bacilli in the lung cavity to the presence of oxygen, for they are equally abundant in the purulent secretion of a tuberculous joint."*

Dr. Whipham gives results of examination of 20 cases, and was disposed to think that in acute tuberculosis and in exacerbations of the disease the bacilli were especially numerous in the sputa, and that when the disease is arrested, or the patients improving, the organisms were greatly reduced in number or disappeared from the expectoration.†

In the discussion which followed the reading of the above mentioned paper at the Medical Society of London, Dr. Samuel West gave his own observations and the following deductions:—

"1. He had found bacilli present in every case of phthisis which he had examined, though in some cases they were in such small numbers as only to be found after repeated and very careful examination.

"2. The number he had found, as a general rule, to vary with the rate of breaking down of the lung, and therefore, in most cases, with the gravity of the disease.

* *Berliner Klinische Wochenschrift*, Nov. 6th, 1882.
† *Lancet*, Feb. 10th, 1883.

"3. The arrangement of the bacilli in the sputum varied much. At times they were isolated, few in number or many, and very numerous in rapid cases; at other times arranged in groups or masses, and this appeared to be the rule in the most rapid cases.

"4. In some instances the bacilli contained small, bright bodies, which had been called spores, and this seemed to be a common condition in acute cases.

"5. There appeared to be but little variations in the size of the individual bacilli in different cases.

"6. In cavities the bacilli existed in large numbers, and usually in masses. The more cheesy matter, or fluid from a cavity, there was in the expectoration the more bacilli we might expect to find; consequently, in a case of acute tuberculosis, before breaking down of the lung, we should expect to find none."

Dr. Burney Yeo pointed out that the absence of bacilli was as important diagnostically as their presence, and mentioned cases in illustration of this fact.

Dr. C. T. Williams gave results of examination of sputum in 130 cases at the Brompton Hospital. Twenty-one were cases other than phthisis, in no one of these did the sputum contain bacilli. The 109 others were cases of phthisis in its various forms, and in 106 of these the bacilli were found, whilst in the three others it could not be affirmed with certainty that they were absent.

Dr. Heneage Gibbes pointed out the existence of two forms of miliary tubercle. In the reticular form he had only succeeded in finding bacilli in three cases, and these in small numbers distributed throughout the reticulum. In the non-reticular form, the tubercles having no fibrillation, and no ant cells, but consisting of irregular cells in the periphery and a caseous mass in the centre, he had invariably found bacilli in large numbers in the caseous centre. He also pointed out the existence of bacilli even in the smallest tubercles. Again, a lung may be stuffed with tubercle, each one containing thousands of bacilli, and yet the patient will die before the destructive process has gone far enough to cause any of them to be ejected in the sputum.*

D'Espine, of Geneva, believes the bacillus to be of the greatest diagnostic value, but worthless as an element in estimating the prognosis. His conclusions are based on the examination of twenty cases with a positive result, and five cases of emphysena and bronchitis with a negative result.†

Dr. Heron gives the results of examination of sputum in sixty-two cases, all of which presented the usual symptoms which we associate with phthisis, and in all of which the bacilli were observed. He ventures to think that the prognosis of cases of consumption will, for the future, hinge upon the numbers and forms in which the bacilli are found in the sputum. He believes that the most rapidly fatal are those in which the bacilli are seen to be *grouped* in numerous masses throughout the sputum.‡

An interesting and important observation, showing the diffusion of bacilli in the air inhaled by patients with tuberculous disease, has been made by Dr. Arthur Ransome. In 1869 the author had examined the aqueous vapour of breath in health and disease. Vapour was condensed in a glass globe, surrounded by ice and salt, and in condensing it was found to carry down all suspended organic matters. On examining the breath of several advanced cases of phthisis, specimens of bacillus were

* *Lancet*, Feb. 24th, p. 321. † *Ibid*, Jan. 13th, 1883.

‡ *Ibid*, Feb. 2nd, 1883.

found in two cases, in several other cases the organism was not found, and it was not found in the aqueous vapour condensed in the waiting-room of the Manchester Consumption Hospital. In order to carry down the organic matter and to afford a basis to attach the material to the cover-glasses, fresh white of eggs, or a little mucus, was added to the fluid. The cover-glasses were treated by Dr. Heneage Gibbes's method of staining.*

This observation is supported by Dr. R. Charnley Smith, who allows the patient to breathe through gun cotton, which is afterwards converted into collodion, and examined for bacilli after the usual process of staining.†

Since my own observations began I have not once failed to detect the tuberculous bacilli in cases which, from other evidence, were assumed to be undoubted ones of tubercular phthisis. On several occasions I have failed to find them on the first examination, and during the cold weather of early winter I obtained only imperfect and unsatisfactory evidence in two or three cases of advanced phthisis at about the same time. One of these (No. 21) soon died, and was shown to be one of rapid tuberculosis by *post mortem* examination; the purulent nummular sputum was repeatedly examined, and only very few ill-defined and ill-stained organisms could be seen, scarcely sufficiently marked to establish their identity. The same result was found in another case at the same time; No. 20 was a case of chronic phthisis, with old cavities, and here I was not able to satisfy myself with the evidence of their presence. My partial failure on those two occasions was no doubt due to the fact rightly pointed out by Dr. Prideaux, of Wellington, that a moderately high temperature is necessary to perfect staining, and since I

* *Brit. Med. Jour.*, Dec. 16th, 1882. † *Ibid*, Jan. 20th. 1883.

adopted the method of warming the staining fluid for the purpose of securing more perfect and more rapid staining, I have been able to obtain the sputum from case No. 20, and found that the bacilli, although not numerous, are very readily and satisfactorily demonstrated.

In a third case, No. 19, I did not at first find bacilli, probably from the same cause (the low temperature of the air), and as the case was one of constitutional syphilis in which lung symptoms had recently manifested themselves, it was of some importance to make subsequent examinations: when this was done, a few weeks later, the bacilli were found to exist in large numbers.

These cases convinced me that no amount of length of exposure of the sputum to the action of the staining fluid would compensate for too low a temperature: the glasses were frequently allowed to remain for twelve and twenty-four hours floating on the stain, and still no bacilli were visible; whereas, if the fluid were treated with a spirit lamp till it began to give off steam, the staining would be rapid and the bacilli would be easily demonstrated.

The whole number of cases I have examined and recorded is 77: of these 49 were one or other of the various forms of tubercular phthisis: the remaining 28 had various other affections of the lung, some of them closely simulating phthisis: the list includes chronic bronchitis, bronchiectasis, chronic syphilitic pneumonia, slight hæmoptysis with no evidence of any disease, chronic pleuro-pneumonia, chronic pleurisy, apex pneumonia with subsequent breaking down from gangrene, sarcoma of lung, grey hepatisation, congestion from mitral disease, diabetes with bronchitis; two cases with strong family history of phthisis, cough with purulent expectoration, but with no evidence of local disease in lungs, the patients rapidly recovering.

Of the whole series of cases there are some few of special interest.

Case 8 was admitted in consequence of sloughing of both tonsils. There was also abundant disease in the lungs. Bacilli were found in the sputum, as well as in the mucus dug out from the cripts of the disintegrating mass of the tonsil. By carbolic spray and antiseptic inhalations the throat gradually got well, and the general health improved. The question arises were the tonsils infected by the passage over them of the mucus containing the bacilli? and this question also suggests another, whether laryngeal disease may not be also a result of local infection from the same cause?

Case 7 was admitted in consequence of chronic diarrhœa, but with little evidence of disease in the lungs. This soon followed, and death took place in six weeks from the first onset of lung symptoms. Post mortem examination was not allowed, but the case appears to be a good illustration of acute pulmonary phthisis arising by infection from a previously diseased intestine.

Case 30 was admitted with symptoms of chronic bronchitis, and no evidences of phthisis were obtained at the first examination. The sputum was found to contain abundant bacilli, and then a more careful examination disclosed the presence of dulness at the right upper lobe. Death took place in two months, the lung having broken down rapidly, and it was found to be riddled with cavities from apex to base. The mucus obtained from the cavities on the post mortem table was found to contain densely crowded masses of bacilli.

Case 39 was one of chronic cystitis of four years duration. Acute pulmonary tuberculosis at last supervened, bacilli were very numerous in the sputum, and a careful examination of the urine disclosed the presence in it of similar organisms. This case illustrates the occurrence of primary disease in urethra and bladder, possibly due to the introduction of bacilli, culminating at last in general tuberculosis.

Of the series of cases in which no bacilli were found:—

Case 2 was one of chronic syphilitic disease of lungs, with evidences of cavity in the upper part of left lower lobe. A few months before, a drainage tube had been passed into this cavity through the chest wall, and the subsequent progress of the patient had been very satisfactory. He again recently came under observation with bronchitis and much muco-purulent sputum, but no bacilli could be detected, and the patient rapidly improved under treatment with iodide of potassium and iodoform.

A series of cases of special interest are the following:—The three patients belong to a family of thirteen, with a slight phthisical history. One sister died from acute phthisis, a brother died from acute pneumonia, and a second from some lung disease. Case No. 5 is that of the brother, 30 years of age, who was laid up in January, 1882, with acute pleurisy; he was then said to have pleuro-pneumonia, and afterwards acute phthisis. When I saw him in May, 1882, he had evidence of chronic pleuro-pneumonia of the right side, with a suspicion of cavity at the upper part of lower lobe. There was no evidence of disease at either apex. The nails were clubbed, and he was anæmic, but had no temperature, and only complained of dyspnœa on exertion. There had been abundant expectoration of muco-pus, which had commenced suddenly in February, and had continued more or less freely.

In December, 1882, he again came under observation, now with some slight hepatic enlargement, and a trace of albumen in urine. Through the winter he expectorated, at times very freely, but I was never able to find any tuberculous bacilli. In April he left Clifton much improved in general condition, having gained weight, having no expectoration, with no albumen in urine, no enlargement of liver, and with no evidence of anything more than chronic pleuro-pneumonia of right lung. The absence of tuberculous bacilli from the sputum quite supports the evidence derived from physical signs, that the disease is and has been a simple inflammatory one, and that its tendency is to cause waxy degeneration of the viscera from chronic suppuration rather than the usual results of pulmonary phthisis.

This patient's sister, case No. 6, is of a similar character. She is 26 years of age, has had chronic pleurisy of left side for two years, is well nourished, but anæmic, and complains of dyspnœa on exertion. There is no decided dulness on percussion, the breath sounds are feeble, crepitation can be heard all down the left side both in front and behind. There is a little non-purulent expectoration, containing putrefactive but no tuberculous bacilli. The physical signs are now at least suggestive of chronic phthisis, but the whole history of the case is that of chronic pleurisy.

The third case of the series, No. 32, is another sister, 24 years of age, who after six months of phthisical dyspepsia and debility came under my observation early in February, 1883. She was found to have cavernous breathing at the left supra spinous fossa, crepitation at the right apex posteriorly and below both clavicles down to the third rib. She had much emaciated, and voice was weak. There was a decided hectic flush, and she had copious night sweating. Expectoration was free, consisting of muco-pus, which was found to be crowded with tuberculous bacilli.

Another sister in the same family was said to be dying of phthisis ten years ago, but is now living and in good health, with no evidence of any lung weakness.

We have here in the same family two sisters and a brother, all with chronic lung disease, two of them of an inflammatory, the other of a typical tuberculous character, the sputum of the latter crowded with bacilli, whilst that of the two former contains none. The phthisical sister and the non-phthisical brother are now living in the same house, and the bacilli are no doubt disseminated freely throughout the house in spite of antiseptic inhalations and disinfection of sputum. The brother, debilitated by chronic pleurisy and chronic suppuration in lung, affords in all probability an excellent field for the growth of bacilli. It will not be surprising if in spite of all precautions his purulent sputum becomes a nidus for the growth of the tuberculous germs, should he escape the perils associated with waxy disease which now threaten him. May not this be the reason why so many cases of chronic pleurisy ultimately die of phthisis, that they become fit subjects for the growth of these all-prevalent tuberculous germs?

The most important observations as to the diagnostic value of the tubercle bacilli have as yet related to the sputum discharged from the air passage. They have, however, been detected in the urine on several occasions.

Rosenstein* has demonstrated their presence in a case of uro-genital tuberculosis of four years duration, and

* *Centralblatt f. d. Med. Wiss.*, 1883, No. 5.

Lichtheim had previously observed them in the pelvis of the kidney of a patient who had died from the same disease. Burney Yeo also states that they have been observed by Barrow in the urine. One of my cases (No. 39) is another illustration of their occurrence in the urine in chronic cystitis, which at last culminated in acute miliary tuberculosis of the lungs, the bacilli being present in the urine and in the sputum. The discharges from the intestine of this patient were not examined for bacilli, but in all probability they would have been found if sought for. They have previously been observed by Lichtheim in the stools of tuberculous patients.*

As regards the examination of fæces, Dr. H. Menche† holds that the presence of the true tubercle bacillus in the intestinal contents is due to the admixture of the secretions of tuberculous ulcers, and that the bacilli are found only in the stools of the really tuberculous. It has been asserted that similar bacilli in the fæces stain in the same way, and are undistinguishable therefore from the bacilli of tubercle. I have myself examined the stools of only two or three cases, in one of these I found numerous tuberculous bacilli, case No. 48 ; this was a case of chronic diarrhœa, with phthisis, in which the sputum contained bacilli. In another patient, with chronic diarrhœa from carcinoma of liver, I found numerous putrefactive bacteria, but no bacilli which would be recognised as like those of tubercle. Further observations are necessary to establish the value of the presence of tubercle bacilli in the fæces as an aid to diagnosis, and, as pointed out by Lichtheim, single staining is better than double staining for this purpose, as in this way the other micro-organisms remain invisible.

* *Fortschritte der Med.*, No. 1, 1883.

† *Vertr. im Niederrhein Ver. f. Nat. a. Heilk,* Jan. 22nd, 1883.

My observations quite accord with those of Ehrlich, Balmer and Fræntzel, Guttmann, D'Espine, Lichtheim, Fränkel, *Z*iehl, Heron, Gibbes, Green, West, Yeo, Whipham, Councilman, and no doubt many others. All agree that while the bacilli are found in the sputum in a very large majority of cases of pulmonary tuberculosis, they are not found in any other disease.

The observation of Koranyi, that he found similar organisms in a case believed to be one of pulmonary syphilis, will be scarcely considered as evidence to the contrary. Three of my cases might have been diagnosed as pulmonary syphilis, inasmuch as all had evidences of lung disease associated with recent constitutional syphilis. Two of these were found to have abundance of tuberculous bacilli in the sputum, and are running the course of ordinary chronic tubercular phthisis, not modified by antisyphilitic treatment; the third is one of chronic lung disease, in which no bacilli are present in the sputum, and which is much benefited by iodide of potassium. The presence of bacilli may, therefore, serve as an indication for treatment, if present iodide of potassium is not likely to be of service, if absent then the disease may be of syphilitic origin, and require treatment accordingly.

Dr. Koch's assertion of the association of the bacillus with tuberculosis, its presence in every case of this disease, its absence in all other morbid conditions, is thus being confirmed by all who investigate it.

His assertion of the causal relation of the parasite to the process stands as yet unchallenged. In spite of outcries from the Western States that the bacillus is only a crystal, every observer who is competent to judge finds that Koch's statement with regard to it cannot be refuted. It is only fair to infer that his further statement, "based upon a demonstration unexcelled in the history of experimental science for accuracy, clearness and completeness" (Dr. W. T. Belfield, Chicago), that the bacillus is the actual cause, the one invariable and essential element in the production of tuberculosis, will be also confirmed by those who have time and opportunity to undertake it. It will be remembered that by a process of cultivation extending over many months, with the isolated descendants of the bacilli found in tuberculous tissues, Koch inoculated numerous animals—using over two hundred altogether—and in every case tuberculous bacilli were found in the infected animal.

If the observations be accurate there is only one conclusion—that these bacilli cause tuberculosis.

*The Cases are epitomised in two classes: the one in which Tuberculous Bacilli were found, the other in which they were believed to be absent.*

CASES IN WHICH TUBERCULOUS BACILLI WERE FOUND:—

| No. | Name, Age, &c. | Condition of Lung. | Bacilli. |
|---|---|---|---|
| 1. | Miss J. T., 40 years ... ... ... | Chronic Phthisis, 3 years. Much purulent expectoration, cavities both apices. | Abundant. |
| 2. | Lydia L., 32 years ... Ward 2 | Paraplegia. Patient wasting and anæmic. Cough, with purulent expectoration. Dulness and cavernous sounds at one apex. | Abundant. |
| 3. | Miss C. C., 20 years ... ... ... | Acute Phthisis. Cough 6 months. Rapid wasting, much purulent expectoration. Cavities in both upper lobes. | Very abundant. |
| 4. | Mr. A. S., 35 years ... ... ... | Early Phthisis, 9 months. Much consolidation of left lung, no breaking down, no pyrexia. Little expectoration. | Abundant. |
| 5. | Mr. F. L., 23 years ... ... ... | Repeated attacks of slight Hæmoptysis. General health good. Small patch of dulness with harsh breathing below right clavicle. Little expectoration. | Numerous. |
| 6. | Mary C., 20 years ... ... Ward 3 | Chronic Phthisis, with history of Syphilis. Cough, night sweats, aphonia, crepitation and dulness at both apices. | Found, but not very numerous. |
| 7. | Alice P., 43 years ... ... Ward 4 | Chronic Diarrhœa. High temperature, rapid breaking down of both lungs. | Very abundant. |

| No. | Name, Age, &c. | Condition of Lung. | Bacilli. |
|---|---|---|---|
| 8. | Alice E., 25 years ... ... Ward 4 | Chronic Phthisis, 18 months, and Sloughing Tonsils, 6 weeks. | Found in sputum and in mucus from recesses of tonsils. Three months afterwards less numerous, only three or four in a field, and this after prolonged antiseptic inhalations. Tonsils quite well and small. |
| 9. | Mary H., 22 years ... ... Ward 1 | Early Phthisis. Small patch of consolidation at one apex. No wasting or temperature. | Numerous. |
| 10. | Henry G., 41 years ... Ward 5 (Dr. Waldo). | Chronic Phthisis. Numerous cavities. ... ... ... | Abundant in lung tissue at P.M.E. |
| 11. | S. B. ... ... ... ... Out-patient | Chronic Phthisis ... ... ... ... ... ... ... .. | Abundant. |
| 12. | Alice P., 25 years ... ... Ward 2 | Chronic Phthisis, with cavities ... ... ... ... ... | Abundant. |
| 13. | Henry M., 16 years ... Ward 7 | Advanced Phthisis ... ... ... ... ... ... ... ... | Abundant. |
| 14. | Henry L., 33 years... ... Ward 7 | Early Phthisis, 3 months. Dulness right upper lobe | Abundant. |
| 15. | Ann J., 42 years ... ... .. ... | Chronic Phthisis ... ... ... ... ... ... ... ... | Present. |
| 16. | Thirza W., 55 years ... Ward 4 | Chronic Phthisis, with Diabetes ... ... ... ... ... | Present, not numerous. |
| 17. | — Morris (Insane patient) ... ... | Chronic Phthisis .. ... ... ... ... ... ... ... | Large, numerous, but isolated. |
| 18. | Arthur A., 9 years ... ... ... | Acute Phthisis, 3 months. High temperature ... | No expectoration voluntarily, but when obtained bacilli found. |
| 19. | Wm. W., 20 years ... ... Ward 6 (Dr. Shaw). | Syphilis and early Phthisis. Not improved by Iodide of Potassium. | Abundant. |
| 20. | John F., 40 years ... ... Ward 5 | Hæmoptysis, 14 years. Cough, 4 years. Emaciation. Cavity at left apex, consolidation at right. Disease not progressive. | Few, mostly small. |

| No. | Name, Age, &c. | Condition of Lung. | Bacilli. |
|---|---|---|---|
| 21. | Emily G., 22 years ... ... Ward 1 | Rapid Phthisis, post partum. Death after 6 weeks' illness. Cavity in right apex, copious disseminated tubercles. | Imperfect evidences from defective staining. |
| 22. | — Brockley ... ... Out-patient | Chronic Phthisis ... ... ... ... ... ... . . ... | Numerous. |
| 23. | Rev. J. K., 40 years ... ... ... | Small patch of dulness at one apex. Feels quite well and at work. | Abundant. |
| 24. | John C., 44 years ... ... Ward 6 | Chronic Phthisis, 4 years ... ... ... ... ... ... | Few. |
| 25. | Wm. W., 25 years ... ... Ward 5 | Chronic Phthisis, 9 years ... ... ... ... ... ... | Present, but not numerous. |
| 26. | Michael D., 50 years ... Ward 7 | Chronic Cough, wasting, 4 years... ... ... ... ... | Very few. |
| 27. | Maria B., 18 years .. ... ... ... | Chronic Phthisis, 4 years. Dulness at one apex ... | Abundant. |
| 28. | Lewis S., 26 years ... ... Ward 7 | Ill four months. Cough, wasting, diarrhœa. Dulness whole of left side. Died in two weeks. | Numerous. |
| 29. | — Taylor . . ... ... Out-patient | Phthisis, 6 months ... ... ... ... ... ... ... ... ... | Abundant. |
| 30. | James L., 42 years ... ... Ward 7 | Case simulating Chronic Bronchitis. Re-examination of chest showed dulness at right upper lobe. Pyrexia and rapid breaking down ensued in spite of antiseptic inhalations. | Sputum found to contain numerous bacilli. Six weeks later bacilli as numerous as before. P. M. E.—Lung riddled with cavities, mucus in them crowded with bac. in groups and masses. |
| 31. | Mark W. ... ... .. Out-patient | Chronic Phthisis ... ... ... ... ... ... ... ... | Abundant. |
| 32. | Annie K., 24 years... ... ... ... | Acute Phthisis. Phthisical Dyspepsia, 6 months. Rapid wasting, breaking down at both apices. | Very numerous. |
| 33. | Emma H., 48 years ... Ward 2 | Chronic Phthisis, 5 years. No active symptoms ... | Not numerous, small and ill-formed. |

| No. | Name, Age, &c. | Condition of Lung. | Bacilli. |
|---|---|---|---|
| 34. | Charles A., 29 years ... Ward 6 | Bronchitis. Pyrexia ensued, and condensation at right apex; afterwards severe tonsillitis, much benefited by iodoform. Expectoration ceased entirely and patient gained in weight. | Sputum found to contain bacilli, not numerous but large. No bacilli in last specimen of sputum, apex clearing. |
| 35. | Wm. C. ... ... ... ... Ward 8 | Chronic Phthisis ... ... ... .. ... ... ... ... | Abundant. |
| 36. | T. H.... ... ... ... . ... ... | Chronic Phthisis ... ... ... ... ... ... ... ... | Abundant and large. |
| 37. | — Thomas, 23 years ... ... ... | Chronic Phthisis ... ... ... ... ... ... ... ... | Decided, but not very numerous, large, well formed, occasional groups. |
| 38. | — Hill ... ... ... ... Ward 3 | Chronic Phthisis ... ... ... ... ... ... ... ... | Numerous, in groups. |
| 39. | E. W., 19 years ... ... ... .. | Chronic Cystitis. Acute Pulmonary Tuberculosis... | In urine. Very numerous in sputum. |
| 40. | — Hanks ... ... ... ... ... .. | (Post-mortem examination). Numerous cavities ... | Lining of cavity crowded with groups and large masses. |
| 41. | John W., 30 years ... ... ... ... | Acute Phthisis, 6 months. Cavities ... ... ... ... | Very abundant. |
| 42. | Wm. R., 59 years ... Out-patient | Chronic Fibroid Phthisis. Hæmoptysis, 6 years ago. Cough ever since. Much condensation of left lung. | Numerous. |
| 43. | — Wright... ... ... ... Ward 8 | Chronic Phthisis ... ... ... ... ... ... ... ... | Moderate number. |
| 44. | — Pates ... ... ... ... ... ... | (P.M.E.) Caseous lining of cavity in lungs ... ... | Masses aggregated together. |
| 45. | — Beachim ... ... Out-patient | Chronic Phthisis ... ... ... ... ... ... ... ... | Numerous. |
| 46. | John P., 23 years ... ... Ward 6 | Phthisis, 1 month. Dulness and crepitation at one apex. Improved with Iodoform. | Abundant. |
| 47. | Allan M., 32 years ... Ward 7 | Chronic Phthisis, 11 months. Aphonia, 5 months. Much consolidation of left lung. No temperature. | Not numerous, short and small. |
| 48. | Miss O., 23 years ... ... ... ... | Phthisis, 12 months. No expectoration ordinarily. Severe diarrhœa, 2 months. | Numerous in sputum, and also in fæces. |
| 49. | — Kelly, 45 years ... ... Ward 7 | Albumenuria, degenerated arteries, debility, temperature. Dulness at one apex. | Present, but small and not numerous. |

CASES IN WHICH NO TUBERCULOUS BACILLI WERE FOUND:—

| No. | Name, Age, &c. | Condition of Lung. | Bacilli. |
|---|---|---|---|
| 1. | Margaret L., 48 years .. ... ... | Emphysema, dilated bronchi, cavities, dilated right heart, &c. No tubercles. | None. |
| 2. | James B., 34 years ... ... Ward 7 | Chronic Syphilitic Pneumonia. Cavity in base, drainage tube. Occasional bronchitis. Much muco-purulent sputum. | None. |
| 3. | Lewis J. ... ... ... ... Ward 7 | Chronic Bronchitis ... ... ... ... ... ... ... ... | None. |
| 4. | — Collingbourne ... Out-patient | Said to have Hæmoptysis. No evidence of any lung disease. | None. |
| 5. | Rev. P. K., 30 years ... ... ... | Chronic Pleuro-pneumonia, 12 months. Much dulness at right base, apex sounds normal. Copious purulent expectoration. Nails clubbed. Family history of Phthisis. Occasional Albumenuria. Liver a little enlarged. | None. |
| 6. | Charlotte K., Sister to above, 26 years. | Chronic Left Pleurisy, 2 years. Persistent Cough. No wasting or Hæmoptysis. Slight dulness over whole of left lung, moist sounds over whole of left side. A little non-purulent expectoration. | None. |
| 7. | Wm. R., 58 years ... ... Ward 8 | Apex-pneumonia. Lung breaking down. Cavity. Purulent expectoration. Gangrene. | None. |
| 8. | — Sanders ... ... ... ... ... | Chronic Bronchitis ... ... ... ... ... ... ... ... | None. |
| 9. | Rev. J. B., Father to case of Phthisis No. 27. | Chronic Bronchitis, much muco-pus ... ... ... ... | None. |
| 10. | John S., 32 years ... ... ... ... | Secondary Sarcoma of Lung... ... ... ... ... ... ... | None. |

| No. | Name Age, &c. | Condition of Lung. | Bacilli. |
|---|---|---|---|
| 11. | — Harris | Chronic Bronchitis | None. |
| 12. | A. B. | Case of Pneumonia, grey hepatisation | None. |
| 13. | — Stonelake, 10 years | Bronchitis. Debility | None. |
| 14. | — Hayward, 15 years ... Ward 3 | Mitral Disease. Bronchial mucus. Double Pneumonia | None. |
| 15. | Selina S., 32 years ... Ward 2 | Bronchitis. Rapid convalescence | None. |
| 16. | Harriet S., 32 years ... Ward 4 | Bronchitis, much muco-pus | None. |
| 17. | — Batten | Chronic Bronchitis | None. |
| 18. | Mrs. M., 65 years | Case of Diabetes. Bronchitis. Thick purulent nummular sputum. Speedy recovery. | None. |
| 19. | — Lovell, 35 years ... Ward 2 | Repeated attacks of Bronchitis. Some condensation of left lung. Morbid growth. | None. |
| 20. | — Tucker | Chronic Bronchitis | None. |
| 21. | Miss J. Mac, 17 years | Family history of Phthisis. Pyrexia, much crepitation in both lungs. Copious purulent expectoration. | None. |
| 22. | Miss H. W. | Family history of Phthisis. Chronic cough, purulent expectoration. No dulness. | None. |
| 23. | Maria W., 18 years ... Ward 2 | History simulating Phthisis. Patient rapidly convalesced. | None. |
| 24. | Mr. W., 50 years | Chronic Pneumonia. No expectoration | None. |
| 25. | Frank S., 21 years ... Ward 5 | Bronchitis. Asthma, 9 months | None. |
| 26. | E. H., 44 years ... Out-patient | Bronchitis | None. |
| 27. | Henry Y., 50 years | Chronic Bronchitis | None. |
| 28. | A. B. G., 61 years | Mitral Disease. Cough and expectoration. Patient much emaciated. | None. |

